# General Practice and Digital Methods to Recruit Stroke Survivors to a Clinical Mobility Study: Comparative Analysis

**DOI:** 10.2196/28923

**Published:** 2021-10-13

**Authors:** Katja Reuter, Chang Liu, NamQuyen Le, Praveen Angyan, James M Finley

**Affiliations:** 1 Department of Public Health & Preventive Medicine SUNY Upstate Medical University Syracuse, NY United States; 2 Southern California Clinical and Translational Science Institute Keck School of Medicine of USC University of Southern California Los Angeles, CA United States; 3 Department of Biomedical Engineering University of Southern California Los Angeles, CA United States; 4 Annenberg School for Communication and Journalism University of Southern California Los Angeles, CA United States; 5 Division of Biokinesiology and Physical Therapy University of Southern California Los Angeles, CA United States

**Keywords:** clinical trial, stroke, falls, digital media, social media, advertising, participant recruitment, Facebook, Google, clinical research, research methods, recruitment practices, enrollment

## Abstract

**Background:**

Participant recruitment remains a barrier to conducting clinical research. The disabling nature of a stroke, which often includes functional and cognitive impairments, and the acute stage of illness at which patients are appropriate for many trials make recruiting patients particularly complex and challenging. In addition, people aged 65 years and older, which includes most stroke survivors, have been identified as a group that is difficult to reach and is commonly underrepresented in health research, particularly clinical trials. Digital media may provide effective tools to support enrollment efforts of stroke survivors in clinical trials.

**Objective:**

The objective of this study was to compare the effectiveness of general practice (traditional) and digital (online) methods of recruiting stroke survivors to a clinical mobility study.

**Methods:**

Recruitment for a clinical mobility study began in July 2018. Eligible study participants included individuals 18 years and older who had a single stroke and were currently ambulatory in the community. General recruiting practice included calling individuals listed in a stroke registry, contacting local physical therapists, and placing study flyers throughout a university campus. Between May 21, 2019, and June 26, 2019, the study was also promoted digitally using the social network Facebook and the search engine marketing tool Google AdWords. The recruitment advertisements (ads) included a link to the study page to which users who clicked were referred. Primary outcomes of interest for both general practice and digital methods included recruitment speed (enrollment rate) and sample characteristics. The data were analyzed using the Lilliefors test, the Welch two-sample t test, and the Mann-Whitney test. Significance was set at *P*=.05. All statistical analyses were performed in MATLAB 2019b.

**Results:**

Our results indicate that digital recruitment methods can address recruitment challenges regarding stroke survivors. Digital recruitment methods allowed us to enroll study participants at a faster rate (1.8 participants/week) compared to using general practice methods (0.57 participants/week). Our findings also demonstrate that digital and general recruitment practices can achieve an equivalent level of sample representativeness. The characteristics of the enrolled stroke survivors did not differ significantly by age (*P*=.95) or clinical scores (*P*=.22; *P*=.82). Comparing the cost-effectiveness of Facebook and Google, we found that the use of Facebook resulted in a lower cost per click and cost per enrollee per ad.

**Conclusions:**

Digital recruitment can be used to expedite participant recruitment of stroke survivors compared to more traditional recruitment practices, while also achieving equivalent sample representativeness. Both general practice and digital recruitment methods will be important to the successful recruitment of stroke survivors. Future studies could focus on testing the effectiveness of additional general practice and digital media approaches and include robust cost-effectiveness analyses. Examining the effectiveness of different messaging and visual approaches tailored to culturally diverse and underrepresented target subgroups could provide further data to move toward evidence-based recruitment strategies.

## Introduction

Participant recruitment remains one of the main challenges in conducting clinical research, with many trials failing due to recruiting an inadequate number of study participants [1,2]. This is also true with regard to recruiting people poststroke to clinical research studies [3]. The disabling nature of a stroke, which often includes functional and cognitive impairments, makes recruiting patients particularly complex and challenging [3,4]. There is generally no centralized system through which researchers can contact people poststroke once they have completed rehabilitation.

More than 795,000 people in the United States experience a stroke every year [5], and those who recover are often left with a range of impairments affecting cognitive and motor function [6,7]. Because of the persistence of these impairments and their influence on the patients’ quality of life, many research groups are developing a range of novel interventions to enhance poststroke recovery [8-11]. Clinical trials are considered the gold standard for evaluating the effects of interventions on health-related biomedical or behavioral outcomes [12]. However, older adults, defined as 65 years of age and older [13], despite being at the highest risk of stroke, have been identified as difficult to reach and are commonly underrepresented in health research, particularly clinical trials [14-16]. One potential reason for the underrepresentation of older adults in clinical trials is that they may not be aware of ongoing trials for which they may be eligible [17]. Digital media and social media (SM) may provide a cost-effective approach to complementing existing recruitment issues and increase the enrollment of stroke survivors in research.

The social networks Facebook and Twitter are among the most popular platforms used for research recruitment [18,19]. A smaller subset of studies has experimented with search engine marketing on Google [20-24] and other search engines, such as Yahoo and Bing, to recruit to studies [25]. Today, a growing body of literature reports the successful use of digital media to recruit older adults. Langbaum et al [26], for example, referred cognitively healthy adults aged 55-75 years in the United States online into a registry of Alzheimer’s disease prevention studies (GeneMatch). Over half of those participants (45,210/75,351, 60%) joined GeneMatch via SM advertisements (ads). Another study reported dwindling participant recruitment and later showed a significant increase in the recruitment of middle-to-older-aged people into a blood pressure randomized controlled trial after implementing a Facebook advertising campaign [27]. Lam and Woo [28] demonstrated the cost-effectiveness of Facebook to recruit elder Chinese-speaking Americans into a health education study. However, there is little evidence of using digital media and advertising for recruiting stroke survivors.

The objective of this case study was to compare the effectiveness of general practice (traditional) and digital methods (online) of recruiting stroke survivors to an under-enrolling clinical study of factors affecting fall risk during walking. In this study, general practices included calling individuals listed in an institutional review board (IRB)–approved stroke registry, contacting local physical therapists, and placing study flyers; digital methods included ads on Facebook and Google search pages. The primary outcomes of interest for both conventional and digital methods included recruitment speed (enrollment rate) and sample representativeness. To support related recruitment efforts by other research teams, we also shared the recruitment strategy and materials.

## Methods

### Study Overview

The clinical mobility study, which began in July 2018, was designed to identify factors that impact balance and fall risk in people poststroke and determine how manipulating these factors influences self-reported perceptions of walking quality and the ability to recover from experimentally imposed loss of balance. The accrual goal was 40 participants. The study required a single visit to a research lab based on a university medical campus in Los Angeles and involved video-based recording of the participants’ walking patterns. After providing informed consent and completing a screening questionnaire to determine eligibility, participants completed a set of clinical assessments. These included the lower-extremity portion of the Fugl-Meyer Assessment, which quantifies motor impairment; the Berg Balance Scale (BBS) [29]; the Functional Gait Assessment [30]; a 10-m walking test; the Activity-Based Confidence Scale [31]; and a fall efficacy and fall history questionnaire if they had experienced at least 1 fall within the past year.

The participants then completed 4 walking trials on a dual-belt treadmill. They first walked on the treadmill for 2 minutes at their self-selected speed. The participants then completed 3 subsequent tests, where they were asked to modify their step lengths to match the lengths of visual targets displayed on a screen in front of the treadmill. In each test, the participants responded to rapid accelerations of the treadmill belts, which acted as perturbations of the participants’ walking patterns. The participants were compensated at a rate of $20 per hour for the time that they spent in the lab. The trial was approved by the IRB of the University of Southern California (USC; HS-18-00417-AM002).

To support research participant recruitment, researchers in the lab where the research was conducted relied on a preexisting participant registry and word-of-mouth referrals from local physical therapists to reach out to stroke survivors who might like to participate in new studies. However, this strategy often had a relatively low rate of return. As of March 2019, with 4 months left in the award period, this study was behind its enrollment target of 40 participants, having recruited only 15 participants. Missing this enrollment target would have resulted in an underpowered study and wasted resources. Therefore, the team decided to include digital recruitment methods on Facebook and Google during the latter portion of the study in 2019.

### Study Population

Eligible study participants included individuals 18 years and older who had a single stroke and were currently ambulatory in the community. Inclusion criteria included (1) a unilateral brain lesion from a single stroke; (2) paresis confined to one side; (3) ability to walk on a treadmill for 2 minutes; (4) absence of cognitive impairment, as demonstrated by a Mini-Mental State Examination score greater than 24; and (5) ability to provide informed consent.

### Recruitment Methods

#### General Practice Recruitment

The standard recruiting practice in the lab was to call individuals listed in an IRB-approved stroke registry or contact local physical therapists affiliated with USC or the Rancho Los Amigos National Rehabilitation Center. Flyers for the study (see Multimedia Appendix 1 for an example) were physically placed throughout USC’s Health Sciences Campus.

#### Digital Recruitment

**Figure 1:**
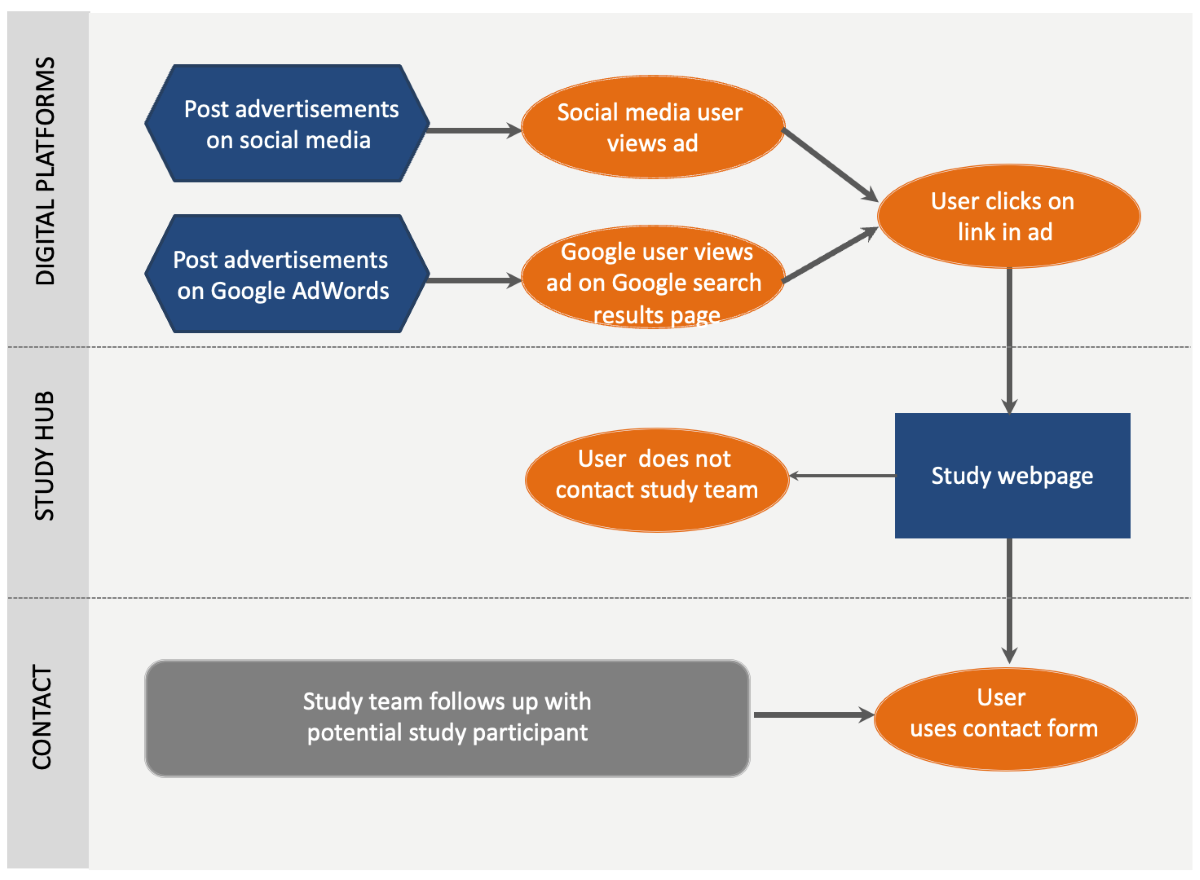
Digital recruitment process for the social network Facebook and Google search results pages.

Between May 21, 2019, and June 26, 2019, the study was promoted digitally using the social network Facebook and the search engine marketing tool Google AdWords. The recruitment ads (Multimedia Appendix 2) were posted on Facebook and Google search results pages. The recruitment ads included a link to the study page (Multimedia Appendix 3) to which users who clicked were referred. On the study page, interested users could use the contact form to get in touch with the study team for further information and screening. Figure 1 outlines the digital recruitment process.

The text and images of the ads were reviewed and approved by the Health Sciences Campus IRB of USC (HS-18-00417). The text and images (Multimedia Appendix 2) were tailored to people of all ages who had a stroke using a mix of images, such as infographics and photos. We did not target the ads using any other characteristics of potential viewers. The type of ad we used per platform and targeting criteria are listed in Multimedia Appendix 4.

### Outcome Measures

The primary outcomes of interest for both conventional and digital methods included recruitment speed (enrollment rate) and sample characteristics. We captured the number of participants enrolled, the time required for enrollment, and participant demographic and clinical characteristics. These measures were used to compute the enrollment rate for each method and to determine differences in the sample representativeness. Secondary outcome measures for our digital recruiting methods included the number of post impressions (ie, the number of times a post was seen without a user necessarily interacting with it), the number of clicks on the link in the message, the number of resulting study page contacts, the number of people screened, the cost per click, and the cost per enrollee.

### Data Collection

Recruitment-related data for each ad were collected in three different ways: (1) SM-based engagement data, such as clicks on the ads and impressions (the number of times an ad was displayed), were collected from Facebook and Google; (2) study website engagement data, including contact requests, were collected using Google Analytics; and (3) enrollment data were provided by the study team. Multimedia Appendix 5 details the technical techniques we used to collect the data from the digital platforms.

### Analysis

Information on recruitment sources was collapsed into two categories: (1) general practice and (2) digital (ie, Facebook and Google). Digital recruitment was further broken down to compare the recruitment results by digital platform. All statistical analyses were performed in MATLAB 2019b (The Mathworks, Natick, USA). We analyzed the normality of participant characteristics, including age, the Fugl-Meyer Assessment score, and the BBS score, using the Lilliefors test. Normally distributed data were expressed as the mean (SD) of the corresponding mean, and we compared the participant characteristics between groups (ie, recruited via general practice or digital approaches) using the Welch two-sample *t* test. Nonnormally distributed data were expressed as the median (IQR). We used the Mann-Whitney test to compare between groups. Significance was set at the *P*=.05 level.

## Results

### Participant Demographics

Overall, the participants (N=40) included 15 (37.5%) women and 25 (62.5%) men, and were aged 29-78 years (mean 59, SD 12 years) (Table 1). This age range is consistent with the current literature, which indicates that stroke is most prevalent in adults aged 60 years and older [32]. Most of the recruited participants were non-Hispanic, that is, Asian (12/40, 30%), followed by one-quarter Hispanic (10/40, 25%), white (9/40, 22.5%), more than one race (9/40, 22.5%), and African American/black (8/40, 20%).

**Table 1:** Characteristics of enrolled study participants by recruitment method.

Characteristic			Recruited through general practice (n=31), n (%)		Recruited through digital media (n=9), n (%)		Total recruited (N=40), n (%)
**Age**							
	18-29 years	1 (3.2)		0 (0)		1 (2.5)	
	30-39 years	2 (6.5)		0 (0)		2 (5.0)	
	40-49 years	3 (9.7)		3 (33.3)		6 (15.0)	
	50-59 years	9 (29.0)		1 (11.1)		10 (25.0)	
	60-69 years	11 (35.5)		4 (44.4)		15 (37.5)	
	70-99 years	5 (16.1)		1 (11.1)		6 (15)	
**Sex**							
	Male	21 (64.5)		4 (44.4)		25 (62.5)	
	Female	10 (35.5)		5 (55.6)		15 (37.5)	
	Other	0 (0)		0 (0)		0 (0)	
**Ethnicity/racial background**							
	African American/Black	6 (19.4)		2 (22.2)		8 (20.0)	
	American Indian/Alaska Native	0 (0)		0 (0)		0 (0)	
	Asian/Pacific Islander	7 (22.6)		5 (55.6)		12 (30.0)	
	Hispanic	9 (29.0)		1 (11.1)		10 (25.0)	
	Middle Eastern	0 (0)		0 (0)		0 (0)	
	White	8 (25.8)		1 (11.1)		9 (22.5)	
	Other	9 (29.0)		0 (0)		9 (22.5)	

### Results of General Practice Recruitment

Throughout 2018, general practice recruitment proceeded slowly as most of the members of the stroke registry who were contacted either did not return the phone calls or were unable to participate in the study. Between July 1, 2018, and May 20, 2019 (46 weeks), we recruited 26 participants through our standard recruiting procedures. Between May 21, 2019, and July 16, 2019 (8 weeks), we recruited an additional 5 participants through general practice recruiting methods. Of these 31 participants, we recruited 20 participants from the existing registry and 11 through word-of-mouth referrals from local physical therapists and participants. The mean age of those recruited through general practice was 58.7 (SD 11.9) years. 

### Results of Digital Recruitment

**Figure 2:**
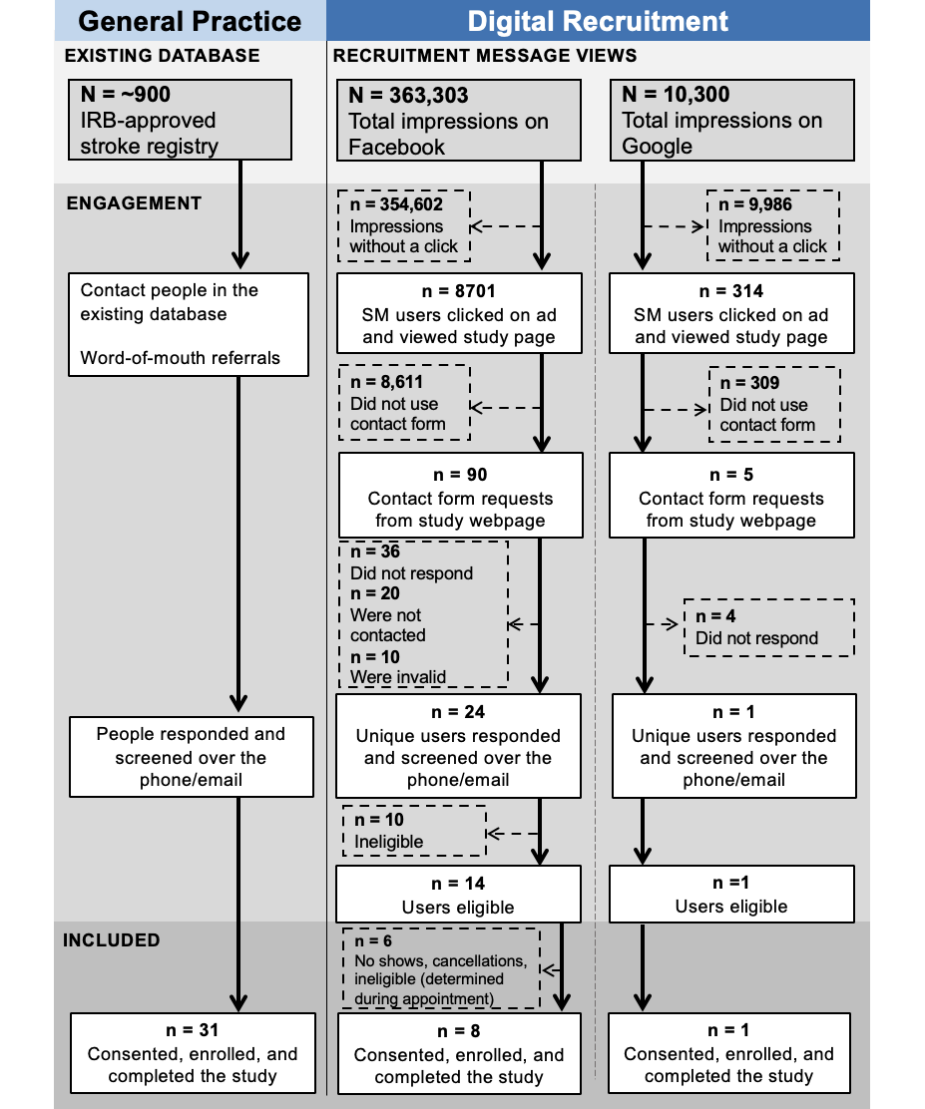
Recruitment flow diagrams for general practice and digital recruitment using Facebook and Google. IRB: institutional review board; SM: social media.

A total of 8 advertised messages were posted (6 on Facebook and 2 on Google) over 5 weeks (between May 21, 2019, and June 26, 2019) (Table 2). Figure 2 shows the recruitment flow diagrams for Facebook and Google. The combined digital recruitment efforts on Facebook and Google resulted in a total of 85 valid referred potential study participants, of which 25 (29.4%) contacted the study team using the web-based contact form. Of these 25, 9 participants met the inclusion criteria, enrolled, and completed the study. The mean age of the participants recruited through digital recruitment was 59.0 (SD 10.7) years, comparable to the mean age of the participants recruited through general practice approaches.

**Table 2:** Performance of digital recruitment ads.

Ad run dates			Impressions, n		Link clicks, n		Cost ($)		Study page contacts, n		People screened, n		People enrolled, n		Cost per click ($)		Cost per enrollee ($)^a^
**Digital platform: Facebook**																	
	5/21/2019-6/26/2019	51,435		580		886.43		15		6		4		1.53		221.60	
	5/22/2019-6/26/2019	75,459		1033		1604.13		21		10		2		1.55		802.07	
	5/24/2019-6/26/2019	106,992		2011		1825.5		18		1		0		0.91		—^b^	
	5/24/2019-6/26/2019	10,852		183		226.46		2		0		0		1.24		—	
	5/21/2019-6/30/2019	84,798		1118		1523.89		2		3		1		1.36		1523.89	
	5/24/2019-6/26/2019	33,767		373		537.28		16		3		1		1.44		537.28	
**Digital platform: Google**																	
	05/13/2019-06/03/2019	9473		291		964.42		3		1		1		3.31		964.42	
	05/13/2019-06/03/2019	876		23		50.59		2		0		0		2.20		—	
	Mean	46,706.50		701.50^c^		952.34		9.88		3		1.13		1.36^d^		846.52^e^	
	Total	373,652		5612		7618.70		79		24		9		N/A^f^		N/A	

^a^Values were calculated by dividing the cost by the number of enrollees for each ad. – indicated that no enrollee was recruited through this ad.

^b^Not available.

^c^Means for clicks and engagement values were rounded off to the nearest whole number. Unless otherwise indicated, these were calculated by dividing by the total number of ads (n=8).

^d^This value was calculated by dividing the total cost of the ads for both platforms by the total link clicks.

^e^This value was calculated by dividing the total cost of the ads for both platforms by the number of total enrollees.

^f^N/A: not applicable.

### Most Cost-Effective Digital Recruitment Messages

**Figure 3:**
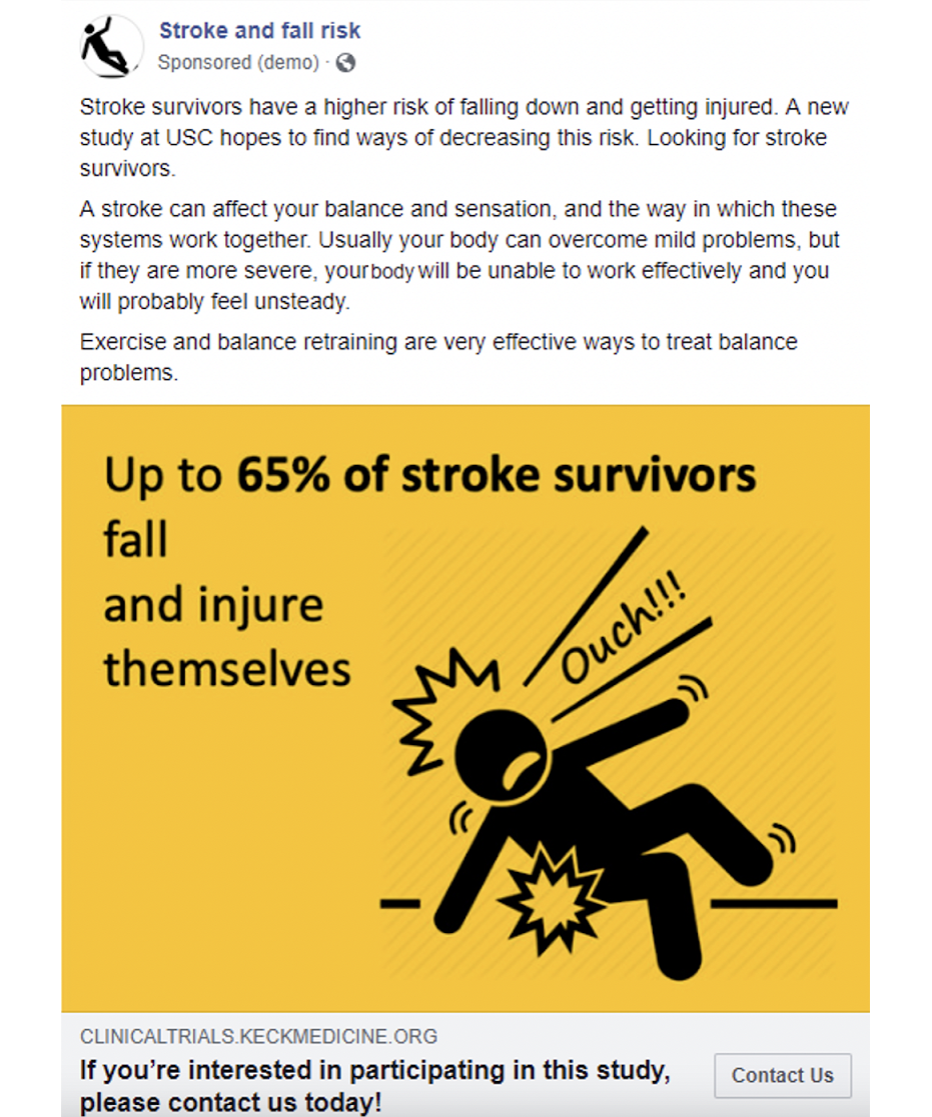
Example of the advertisement that ran on Facebook, with the lowest cost per enrollee.

The digital recruitment ads used different types of images, including infographics, images of older couples, and older individuals after a fall. Figure 3 shows the ad message that ran on Facebook, with the lowest cost per enrollee ($221.60) compared to a higher cost per enrollee on Google ($964.42) (Table 2). Multimedia Appendix 2 shows the recruitment ad messages used in this study. We only ran two simultaneous Google ads for about 3 weeks because of the cost of the ads and the available budget.

### Comparison of Recruitment Methods

We found that the rate of enrollment was higher when using digital media (1.8 people/week) than when using general practice approaches (0.57 people/week). We did not find any significant differences in age, motor impairment level measured by Fugl-Meyer scores, and functional balance measured by the BBS (Table 3). Because of the small sample size, we were not able to statistically compare the outcomes related to gender and race/ethnicity for general practice and digital recruitment methods.

**Table 3:** Comparison of participant characteristics recruited via general practice versus digital approaches.

Characteristics	Recruited through general practice	Recruited through digital media	*P* value
Age (years), mean (SD)	59 (12)	59 (11)	.95
Fugl-Meyer Assessment score, median (IQR)	28 (25-30)	25 (20-29)	.22
BBS^a^, median (IQR)	53 (48-54)	52 (49-54)	.82
Enrollment rate (number of people enrolled/week)^b^	0.57 (31/54)	1.8 (9/5)	N/A^c^

^a^BBS: Berg Balance Scale.

^b^The rate of enrollment was calculated as the total number of people enrolled from each recruiting method/week between the first and last enrollment.

^c^N/A: not applicable.

## Discussion

### Primary Results

Our results indicate that digital recruitment methods can address recruitment challenges regarding stroke survivors. The use of digital approaches allowed us to enroll study participants and close the recruitment gap at a faster rate (1.8 participants/week) compared to using general practice approaches (0.57 participants/week). The effectiveness of SM-based recruitment methods, for example, in comparison to more general (traditional) approaches has been shown by a range of studies [33]. However, comparable data on the effectiveness of recruitment efforts for stroke survivors are currently limited. The characteristics of the enrolled stroke survivors did not differ significantly by age (*P*=.95) or clinical scores (*P*=.22; *P*=.82) between digital and general practice approaches. However, we observed a trend toward recruiting a higher percentage of female participants through digital methods as compared to general practice approaches. Our findings demonstrate that digital and general recruitment practices can achieve similar levels of sample representativeness, with the possibility that digital methods may even result in samples with distributions of sex that are more representative of the general population. There is limited research on the representativeness of participants recruited from digital media and SM. However, although some studies have reported issues related to representativeness and selection bias [34,35], our data support previous findings from other studies that reported the successful use of SM to recruit comparable and representative samples, as described in the systematic review by Whitaker et al [36]. Additionally, Yu et al [37], for example, were able to recruit geographically representative samples of individuals with myeloproliferative neoplasms in the United States for a survey study using multiple recruitment strategies, including Google and Facebook.

Furthermore, comparing the cost-effectiveness of Facebook and Google, we found that the use of Facebook resulted in a lower cost per click and cost per enrollee per ad. The effectiveness (with varying costs) of Facebook as a recruitment mechanism has been reported in a number of studies across disease or health topics and study types [36], such as mental health [38], mobile health studies in psycho-oncology [39], a clinical trial involving healthy elderly [40], and suicide prevention research [35]. Google ads have also been reported as recruitment methods but less frequently [41,42].

### Limitations

The external validity of our findings is limited. Study participants were targeted and recruited in LA County. Therefore, it is unclear to what extent the same recruitment and targeting approach will lead to comparable results in other US regions or internationally. It is also important to note that certain populations are less likely to respond to social media ads. These populations include people who are older, live in rural areas, have little or no access to the internet, have a low level of internet literacy, or do not use SM. Additionally, we were not able to conduct a complete cost comparison between digital and general recruitment approaches. At the time of study setup, the study team did not plan to compare the cost-effectiveness of different recruitment methods and did not collect related data, for example, the percentage effort spent by the study coordinator and the principal investigator on recruitment efforts and the number of potential participants contacted and screened with general practice methods. Lastly, it should be noted that the sample size recruited using SM was small in this study, and thus the statistical comparison of differences in age and clinical scores between recruiting methods should be confirmed with larger samples. Future studies should also determine whether there are meaningful differences in sex or race between participants recruited via conventional and digital methods, as these methods may differ in their ability to identify a diverse pool of potential study participants.

### Conclusion

Digital recruitment can be used to expedite participant recruitment of stroke survivors compared to general, more traditional recruitment practices, while also achieving equivalent sample representativeness. Both general practice and digital media recruitment methods will be important for the successful recruitment of stroke survivors. The data we provide here demonstrate the potential of digital recruitment methods to aid in meeting the accrual goal without delay. Future studies could focus on testing the effectiveness of different digital platforms and include robust cost-effectiveness analyses. Additionally, examining the effectiveness of different messaging and visual approaches tailored to culturally diverse and underrepresented target subgroups could provide further data to develop evidence-based recruitment strategies.

## Appendix

Multimedia Appendix 1 Flyer used for general practice recruiting.

Multimedia Appendix 2 Recruitment messages used for advertising on Facebook and Google.

Multimedia Appendix 3 Study page to which users who clicked digital ads were referred. ad: advertisement.

Multimedia Appendix 4 Type of ad and targeting criteria used on Facebook and Google. ad: advertisement.

Multimedia Appendix 5 Description of technical approaches used to collect the data from the digital platforms.

## Abbreviations

BBS: Berg Balance Scale
IRB: institutional review board
SM: social media
USC: University of Southern California
