# Supplementary material for: General Practice and Digital Methods to Recruit Stroke Survivors to a Clinical Mobility Study: Comparative Analysis
Source: J Med Internet Res. 2021 Oct 13;23(10):e28923. doi: 10.2196/28923 (PMC8552096; doi:10.2196/28923)
Supplement: Multimedia Appendix 1 [file jmir_v23i10e28923_app1.docx]

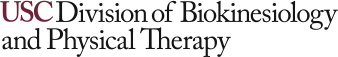
IRB Study #: HS-18-00417

#
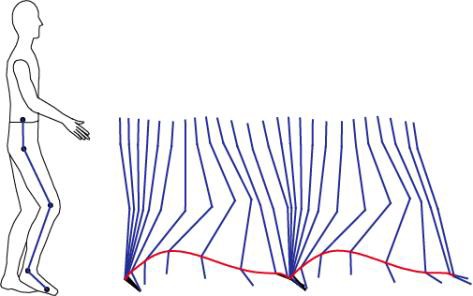
Attention: Research Volunteers Needed Stroke survivors ages 18-85

You are invited to participate in a study to understand how we control balance and walking. The study will take place at the University of Southern California

# Division of Biokinesiology and Physical Therapy

Participation involves having small sticky markers placed on your arms and legs so a computer can detect your movements as you stand, walk on the floor, and walk on a treadmill. We will also quickly test your senses and reflexes with a brief neurological exam.

There are no medical benefits or significant risks to you for joining this study. There are a few different study protocols, each involving a single visit for 2-3 hours, and you may be eligible to participate in more than one protocol. Let us know if you have a time preference and we will be sure to enroll you in a study that works with your schedule.

You will be compensated for participation in this study **For information, contact the Locomotor Control Lab at: XXX-XXX-XXXX or** [**email@email.com**](mailto:email@email.com)

**James M. Finley, PhD, Principal Investigator**

| BKN/PT Walking Study IRB Study #: HS-18-00417  Locomotor Control Lab  XXX-XXX-XXXX  [email@email.com](mailto:email@email.com) | BKN/PT Walking Study IRB Study #: HS-18-00417  Locomotor Control Lab  XXX-XXX-XXXX  [email@email.com](mailto:email@email.com) | BKN/PT Walking Study IRB Study #: HS-18-00417  Locomotor Control Lab  XXX-XXX-XXXX  [email@email.com](mailto:email@email.com) | BKN/PT Walking Study IRB Study #: HS-18-00417  Locomotor Control Lab  XXX-XXX-XXXX  [email@email.com](mailto:email@email.com) | BKN/PT Walking Study IRB Study #: HS-18-00417  Locomotor Control Lab  XXX-XXX-XXXX  [email@email.com](mailto:email@email.com) | BKN/PT Walking Study IRB Study #: HS-18-00417  Locomotor Control Lab  XXX-XXX-XXXX  [email@email.com](mailto:email@email.com) | BKN/PT Walking Study IRB Study #: HS-18-00417  Locomotor Control Lab  XXX-XXX-XXXX  [email@email.com](mailto:email@email.com) | BKN/PT Walking Study IRB Study #: HS-18-00417  Locomotor Control Lab  XXX-XXX-XXXX  [email@email.com](mailto:email@email.com) |
| --- | --- | --- | --- | --- | --- | --- | --- |
