# Supplementary material for: General Practice and Digital Methods to Recruit Stroke Survivors to a Clinical Mobility Study: Comparative Analysis
Source: J Med Internet Res. 2021 Oct 13;23(10):e28923. doi: 10.2196/28923 (PMC8552096; doi:10.2196/28923)

**Multimedia Appendix 2. Recruitment messages used for advertising on Facebook and Google. Numbers of advisements correspond with numbers on Table 2 of the paper.**

#1: Facebook ad:


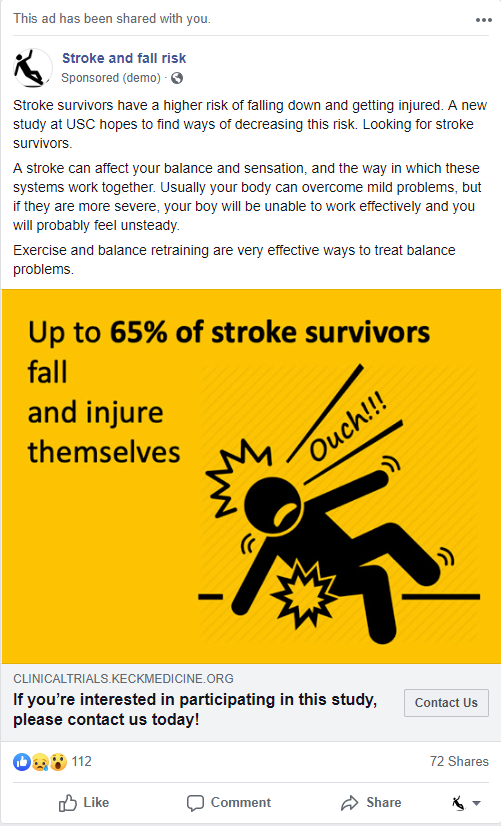


#2: Facebook ad:


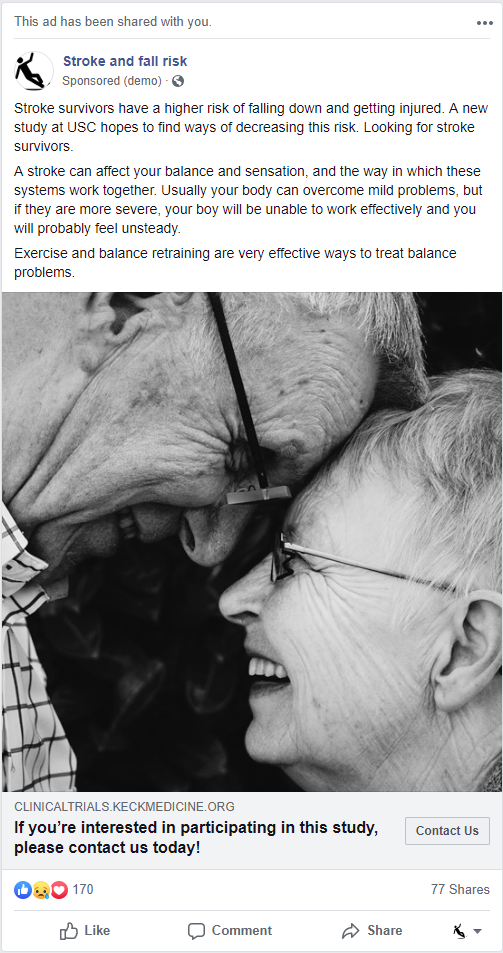


#3: Facebook ad:


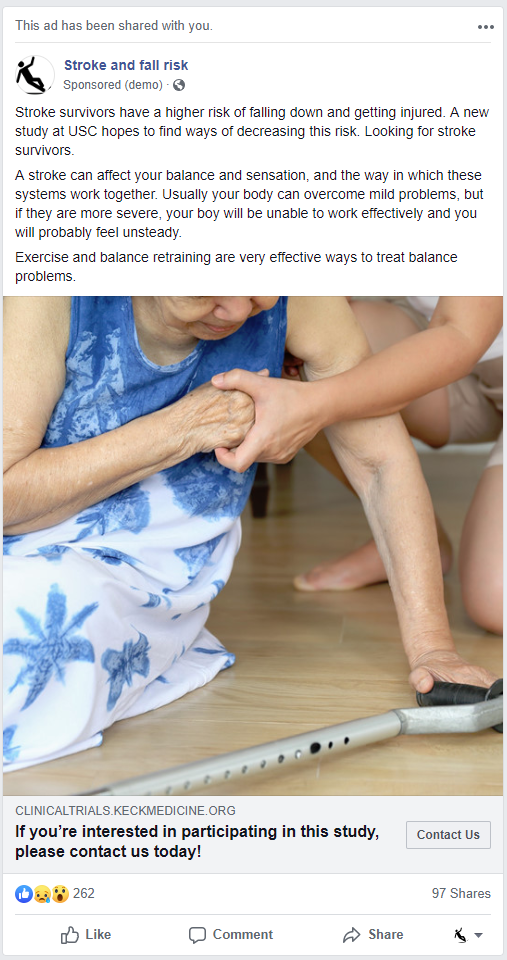


#4: Facebook ad:


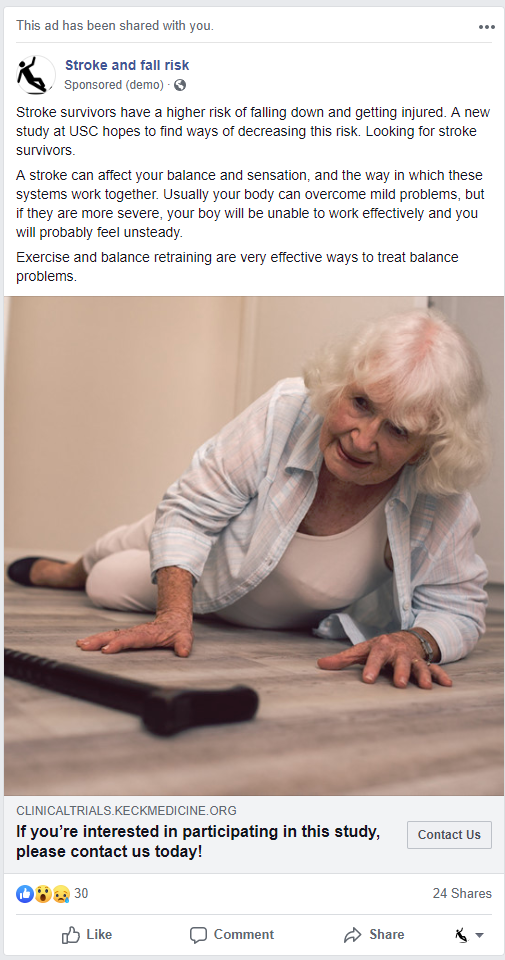


#5: Facebook ad:


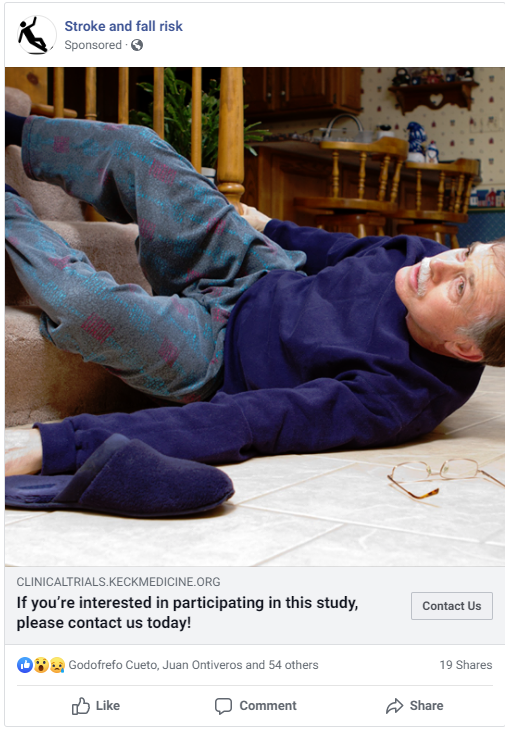


#6: Facebook ad:


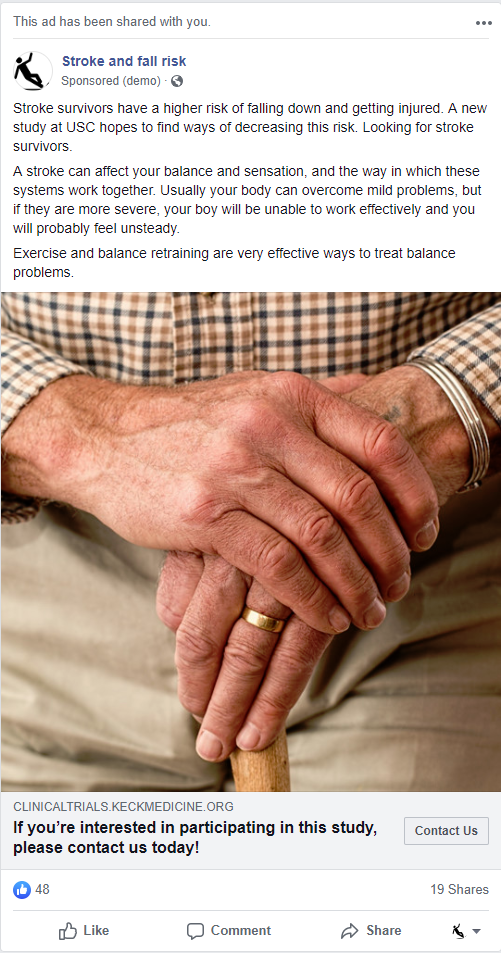


#7: Google ad:


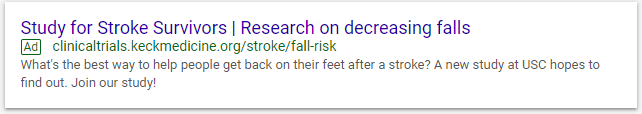


#8: Google ad:


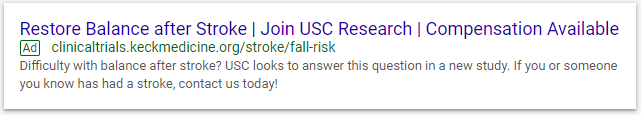

Supplement: Multimedia Appendix 2 [file jmir_v23i10e28923_app2.docx]
