# Supplementary figures and images for: General Practice and Digital Methods to Recruit Stroke Survivors to a Clinical Mobility Study: Comparative Analysis
Source: J Med Internet Res. 2021 Oct 13;23(10):e28923. doi: 10.2196/28923 (PMC8552096; doi:10.2196/28923)

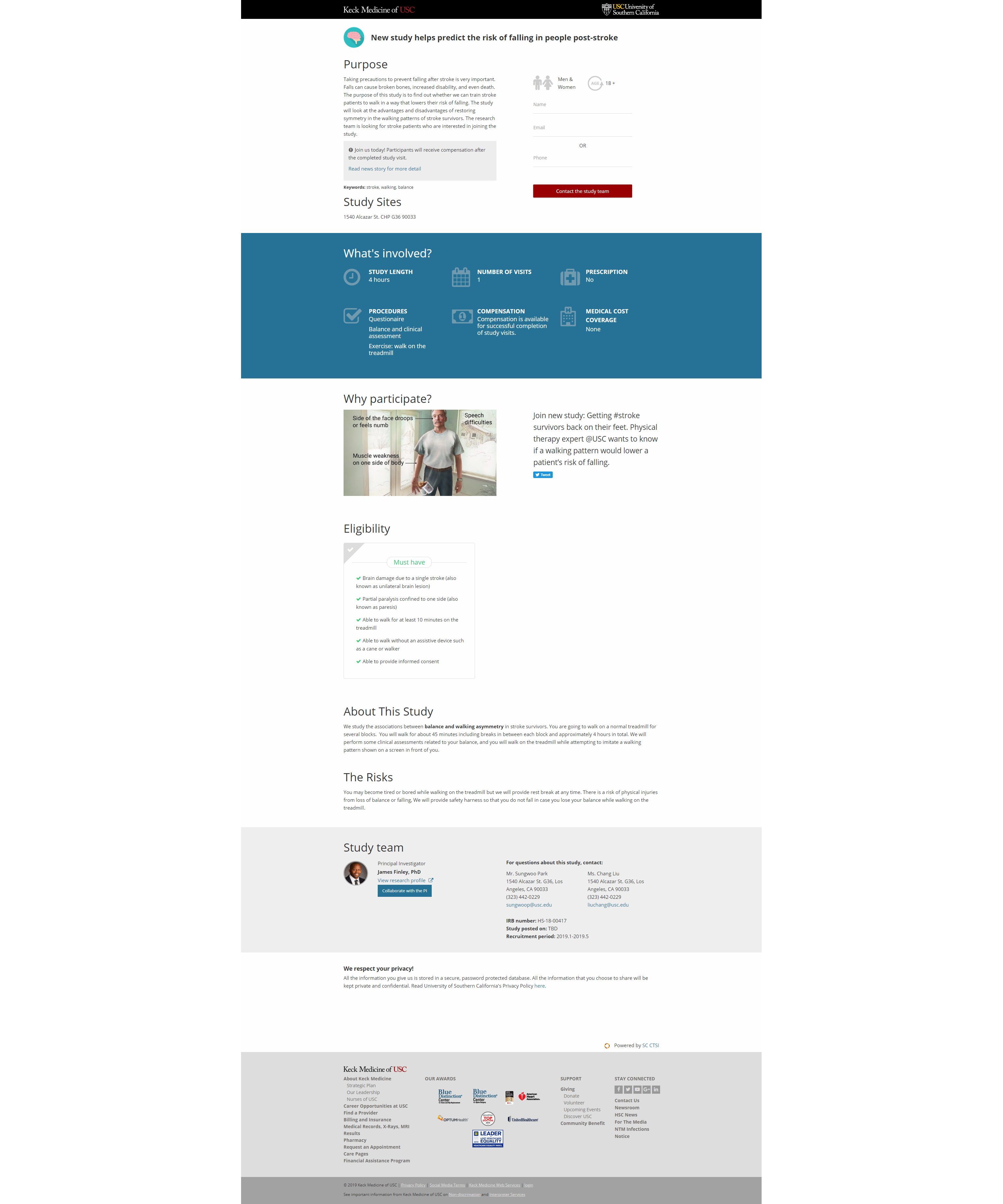

Supplement: Multimedia Appendix 3 [file jmir_v23i10e28923_app3.png]
