# Supplementary material for: General Practice and Digital Methods to Recruit Stroke Survivors to a Clinical Mobility Study: Comparative Analysis
Source: J Med Internet Res. 2021 Oct 13;23(10):e28923. doi: 10.2196/28923 (PMC8552096; doi:10.2196/28923)
Supplement: Multimedia Appendix 4 [file jmir_v23i10e28923_app4.docx]

**Multimedia Appendix 4.** **Type of advertisement and targeting criteria used on Facebook and Google.**

| **Criteria** | **Facebook** | **Google** |
| --- | --- | --- |
| Type of advertisement | Ad that appeared in user’s newsfeeds (while logged in) on all mobile devices | Ad that appeared when user searched for relevant keywords on Google on all mobile devices |
| Targeting | | |
| Location | Los Angeles (+25 mi) California | Los Angeles (+25 mi) California |
| Gender | All | All |
| Age | 18 - 65+ | N/A |
| Language | English (US) | English |
| Keywords | N/A | - Massive stroke - Stroke treatment guidelines - Stroke recovery - Stroke recovery time - Foot drop after stroke - Stroke treatment - Mini stroke - Stroke - Types of stroke - Embolic stroke - Stroke treatment - Signs of aneurysm - Stroke rehab - Ischemic stroke - Aneurysm + brain aneurysm - Paresis - Stroke recovery - Stroke rehabilitation - Brain aneurysm causes - After a stroke - Brain stroke - Fast stroke - Hemorrhagic stroke - Stroke therapy |
| Interests | - Stroke Association - Stroke Awareness | N/A |
| Interest expansion | None | N/A |
