# Supplementary material for: General Practice and Digital Methods to Recruit Stroke Survivors to a Clinical Mobility Study: Comparative Analysis
Source: J Med Internet Res. 2021 Oct 13;23(10):e28923. doi: 10.2196/28923 (PMC8552096; doi:10.2196/28923)
Supplement: Multimedia Appendix 5 [file jmir_v23i10e28923_app5.docx]

**Multimedia Appendix 5. Description of technical approaches used to collect the data from the digital platforms.**

| **Tasks** | **Technical approach** |
| --- | --- |
| Traceable ads | Ads were created based on IRB-approved text message and image. Each ad was given a unique ad name (i.e., identifier) using a combination of numerical identifiers and the name of the study, e.g., 7-stroke-and-fall-risk-post-440. |
| Trackable links for use in ads | Trackable links were created using UTM parameters*. The values used for each UTM parameter are described below. Values were lowercase, special symbols were removed, and all spaces converted to dashes.   - Website URL: Base URL for the study landing page - Campaign Source: Name of digital/social platform (Google/Facebook) - Campaign Medium: Marketing medium (ad/organic) - Campaign Name: Shortened name of study - Campaign Term: Not used - Campaign Content: Ad name (identifier)   Example of a URL with UTM parameters: http://www.study.com/landing-page.html?utm_source=google&utm_medium=ad&utm_campaign=stroke&utm_content=1-stroke-ad-2 |
| Collection of ad performance data | Each digital media platform provided a mechanism to download ad performance data through the ad account including the number of clicks, cost, and impressions per ad.  We used this data to calculate the click-through-rate (CTR: clicks divided by impressions) for each ads and the cost-per-click (CPC: cost per ad divided by total number of clicks on ad). |

* Campaign URL Builder — Google Analytics Demos & Tools. Available from: https://ga-dev-tools.appspot.com/campaign-url-builder/
